# More Diseases Tracked by Using Google Trends

**DOI:** 10.3201/eid1508.090299

**Published:** 2009-08

**Authors:** Camille Pelat, Clément Turbelin, Avner Bar-Hen, Antoine Flahault, Alain-Jacques Valleron

**Affiliations:** Institut National de la Santé et de la Recherche Médicale, Paris, France (C. Pelat, C. Turbelin, A.-J. Valleron); Université Pierre et Marie Curie–Paris 6, Paris (C. Pelat, C. Turbelin, A.-J. Valleron); Université Paris Descartes, Paris (A. Bar-Hen); École des Hautes Études en Santé Publique, Paris (A. Flahault)

**Keywords:** Population surveillance, disease outbreaks, influenza, viruses, France, communicable disease control, letter

**To the Editor:** The idea that populations provide data on their influenza status through information-seeking behavior on the Web has been explored in the United States in recent years (*1*,*2*). Two reports showed that queries to the Internet search engines Yahoo and Google could be informative for influenza surveillance (*2*,*3*). Ginsberg et al. scanned the Google database and found that the sum of the results of 45 queries that most correlated with influenza incidences provided the best predictor of influenza trends (*3*). On the basis of trends of Google queries, these authors put their results into practice by creating a Web page dedicated to influenza surveillance. However, they did not develop the same approach for other diseases. To date, no studies have been published about the relationship of search engine query data with other diseases or in languages other than English.

We compared search trends based on a list of Google queries related to 3 infectious diseases (influenza-like illness, gastroenteritis, and chickenpox) with clinical surveillance data from the French Sentinel Network (*4*). Queries were constructed through team brainstorming. Each participant listed queries likely to be used for searching information about these diseases on the Web. The query time series from January 2004 through February 2009 for France were downloaded from Google Insights for Search, 1 of the 2 websites with Google Trends that enables downloading search trends from the Google database (*5*). Correlations with weekly incidence rates (no. cases/100,000 inhabitants) of the 3 diseases provided by the Sentinel Network were calculated for different lag periods (Pearson coefficient ρ).

The highest correlation with influenza-like illness was obtained with the query grippe –aviaire –vaccin, the French words for influenza, avian, and vaccine respectively (ρ = 0.82, p<0.001). The minus signs removed queries that contained the terms avian or vaccine. Use of the query word grippe alone resulted in a lower correlation (ρ = 0.34, p<0.001). The high double peak in 2005–2006 and the smaller peaks preceding annual epidemics observed with the query word grippe alone were decreased by this specification. However, the unusual double-peak shape of the 2005–2006 epidemic remained (Appendix Figure, panel A).

The highest correlation with acute diarrhea was obtained when we searched for the French word for gastroenteritis (ρ = 0.90, p<0.001). Various spellings were used to account for the presence/absence of an accent or a hyphen. The Google database was searched for gastro-enterite + gastro-entérite + gastroentérite + gastroenterite + (gastro enterite) + (gastro entérite). The + sign coded for or, enabling searches for queries containing >1 of the terms. The second highest correlation was obtained when the keyword gastro (ρ = 0.88, p<0.001) (Appendix Figure, panel B) was used. The highest correlation with chickenpox was obtained with the French word for chickenpox (varicelle) (ρ = 0.78, p<0.001) (Appendix Figure, panel C).

A time lag of 0 weeks gave the highest correlations between the best queries for influenza-like illness and acute diarrhea and the incidences of these diseases; the peak of the time series of Google queries occurred at the same time as that of the disease incidences. The best query for chickenpox had a 1-week lag, i.e., was 1 week behind the incidence time series.

In conclusion, for each of 3 infectious diseases, 1 well-chosen query was sufficient to provide time series of searches highly correlated with incidence. We have shown the utility of an Internet search engine query data for surveillance of acute diarrhea and chickenpox in a non–English-speaking country. Thus, the ability of Internet search-engine query data to predict influenza in the United States presented by Ginsberg et al. (*3*) appears to have a broader application for surveillance of other infectious diseases in other countries.

## Supplementary Material

 Appendix Figure Time series of search queries plotted along the incidence of 3 diseases (influenza-like illness, gastroenteritis, and chickenpox), 2004-2008. Black lines show trends of search fractions containing the French words for influenza (A), gastroenteritis (B), and chickenpox (C). Red lines show incidence rates for the 3 corresponding diseases (influenza-like illness, acute diarrhea, and chickenpox). Search fractions are scaled between 0 and 100 by Google Insights for Search's internal processes (*5*). Incidence rates are expressed in no. cases for 100,000 inhabitants, as provided by the Sentinel Network (*4*).
