# Supplementary figures and images for: More Diseases Tracked by Using Google Trends
Source: Emerg Infect Dis. 2009 Aug;15(8):1327–8. doi: 10.3201/eid1508.090299 (PMC2815981; doi:10.3201/eid1508.090299)

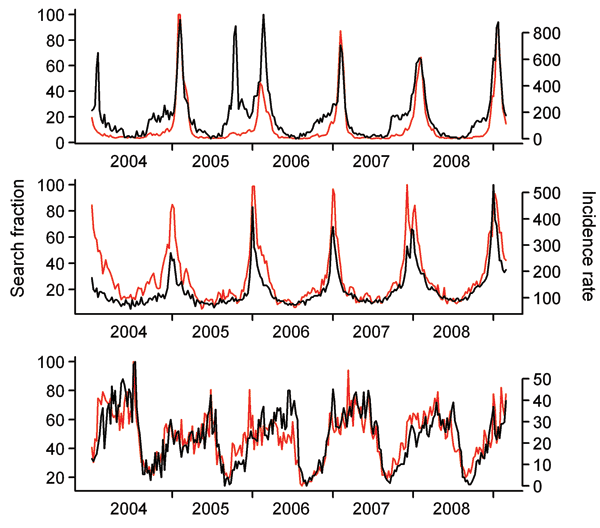

Supplement: Appendix Figure — Time series of search queries plotted along the incidence of 3 diseases (influenza-like illness, gastroenteritis, and chickenpox), 2004-2008. Black lines show trends of search fractions containing the French words for influenza (A), gastroenteritis (B), and chickenpox (C). Red lines show incidence rates for the 3 corresponding diseases (influenza-like illness, acute diarrhea, and chickenpox). Search fractions are scaled between 0 and 100 by Google Insights for Search's internal processes (5). Incidence rates are expressed in no. cases for 100,000 inhabitants, as provided by the Sentinel Network (4). [file 09-0299_appF-s1.gif]
